# Identifying and Responding to Gaps in the Academic Research Pipeline: Findings From the International Society for Autism Research (INSAR) Early Career Committee

**DOI:** 10.1002/aur.70028

**Published:** 2025-03-24

**Authors:** Michele E. Villalobos, Giacomo Vivanti, Subhashini Jayanath, Kimberly Carpenter, Mark D. Shen, Frederick Shic, Jill Locke

**Affiliations:** ^1^ Department of Pediatrics University of Utah Salt Lake City Utah USA; ^2^ A.J. Drexel Autism Institute Drexel University Philadelphia Pennsylvania USA; ^3^ Department of Pediatrics, Faculty of Medicine University of Malaya Kuala Lumpur Malaysia; ^4^ Department of Psychiatry and Behavioral Sciences Duke University Durham North Carolina USA; ^5^ Carolina Institute for Developmental Disabilities, Neuroscience Center, and Department of Psychiatry University of North Carolina at Chapel Hill School of Medicine Chapel Hill North Carolina USA; ^6^ University of Washington Seattle WA USA; ^7^ Seattle Children's Research Institute Seattle WA USA; ^8^ Psychiatry and Behavioral Sciences University of Washington Seattle WA USA

**Keywords:** autism, career development, early career researcher, global training resources

## Abstract

The International Society for Autism Research (INSAR) was established in 2001 with the purpose of providing researchers in the field of autism a specific venue to enhance the quality of multidisciplinary research and elevate the field among the sciences. The autism field and size of the society has now expanded greatly to include over 5000 members, 29% of whom identify as early career researchers. In 2016, a survey was conducted with these early career researchers to identify existing supports and areas of need necessary for a successful and sustained autism research career. Results clearly identified three areas of need: (1) networking opportunities with “change makers,” including funding agencies, journal editors, and senior autism researchers; (2) ensuring early career researchers in the autism research field were diverse and representative of the world; and (3) support from senior researchers to help early career researchers secure their first independent grant and move through promotion. The INSAR Early Career Committee (ECC) was established and developed three initiatives to address the aforementioned gaps: Research Rapid Rounds, the Global Representatives Initiative Pilot (GRIP), and the Mentoring Initiative. The ECC has successfully connected over 34 early career researchers and 40 students/trainees to mentors in 19 unique countries through networking programs and events and has established representatives in seven different countries outside the US and UK. These initiatives are a step towards supporting early career academics in the autism field and bring together autism researchers from around the world to share their work and create professional collaborations to forge our field forward.

**Summary**

- Early career researchers entering the field of autism across the globe identified the need for more support.
- This paper describes the development of an infrastructure to support early career researchers including tiered mentorship, support from senior grant reviewers, and a global representatives program.
- These initiatives provide an effective model for autism programs globally working to support early career stage researchers.

## 1 Introduction

The International Society for Autism Research (INSAR) initiated the Early Career Committee (ECC) in 2016 to support early career researchers in the field of autism to build and support their careers. Early career (EC) is defined by INSAR as “Faculty or non‐academic autism researchers who have completed their formal training (i.e., terminal degree, post‐PhD fellowship or post‐doctoral fellowship, or other specialized training) within the past 10 years.” There is a great need for support for researchers at this career stage to increase retention in academia and ensure that “burn‐out” does not occur.

It is important to view this effort as part of the broader landscape of the need for adequate support for autistic individuals. There is a mismatch between the dire need for autism providers and the limited supply of trained providers, as highlighted by a study on the supply of applied behavioral analysis (ABA) providers in the United States of America (Zhang and Cummings 2020). Continuation of research in autism is necessary to continue to shine a spotlight on key areas of support needs of autistic individuals. The work done by EC researchers is a vital part of this picture. The continued productivity of EC researchers is thus crucial for the field, as they are the future of autism research, and there is a significant attrition rate at this career phase. Availability of funding, work flexibility, and tenure extensions were identified as important types of support desired by EC researchers in a 2021 study (Harrop et al. 2021).

This need also was identified 5 years prior to that, in 2016, when the ECC disseminated a survey to early career INSAR members to identify existing supports and areas of need necessary for a successful and sustained autism research career. The results of the survey were shared and discussed with early career stakeholders during an informal “coffee hour” session at the INSAR 2016 and 2017 annual meetings (see Section 2.2 and Table 1). These surveys and two meetings generated quantitative and qualitative data that support the tremendous need for resources for those moving through the initial stages of their research career. These were largely consistent with previously identified “gaps” in support during this career period (Kupfer et al. 2009, 2016). Namely, EC autism researchers were interested in: (1) networking opportunities with “change makers,” including funding agencies, journal editors, and senior autism researchers—all of whom could provide personalized advice and guidance on creating successful independent research programs and attractive research portfolios for funding; (2) ensuring EC researchers in the autism research field were diverse and representative of the world; and (3) support from senior and established autism researchers to help secure the EC researchers' first independent grant and move through promotion.

The next step was the formation of a new INSAR committee for Early Career members. This committee was closely aligned with INSAR's strategic mission in that it would serve the goal of fostering the next generation of researchers. An application review process over the summer of 2016 was initiated to select committee members from a variety of disciplines representing the majority of EC members based on the results of our survey (e.g., psychology, neuroscience, epidemiology, genetics, biology, education, other). The primary objectives of the INSAR ECC are to: (1) foster communication and collaboration across all areas of autism research (e.g., genetics, behavioral, early identification, brain imaging, and service access) across multiple career levels (e.g., students, early career faculty, and established investigators); and (2) provide supports to EC researchers to ensure their success and longevity in autism research. The INSAR ECC advances these objectives and tenets through the early career events at the INSAR Annual Meeting (i.e., Research Rapid Rounds), the Global Representatives Initiative Pilot (GRIP), and the Mentoring Initiative.

These activities bring together multidisciplinary autism researchers from around the world to share their work and create professional connections and collaborations to forge our field forward. The gap in research output and career support for EC researchers from lower‐ or middle‐income countries (LMIC), compared to upper‐income countries is a reality. Since research generated from LMIC often is not as well‐funded, the output may look dissimilar from that of upper‐income countries. This can lead to a breakdown in communication of the applicability and potential of research findings from LMIC, particularly in empowering communities and researchers. The immense need for equitable research support was highlighted in a recent article (de Vries 2024). The opinion piece stressed the importance of increasing the research capacity of a new generation of researchers, partnership with LMIC communities, as well as mentoring clinical researchers from LMIC.

**FIGURE 1:**
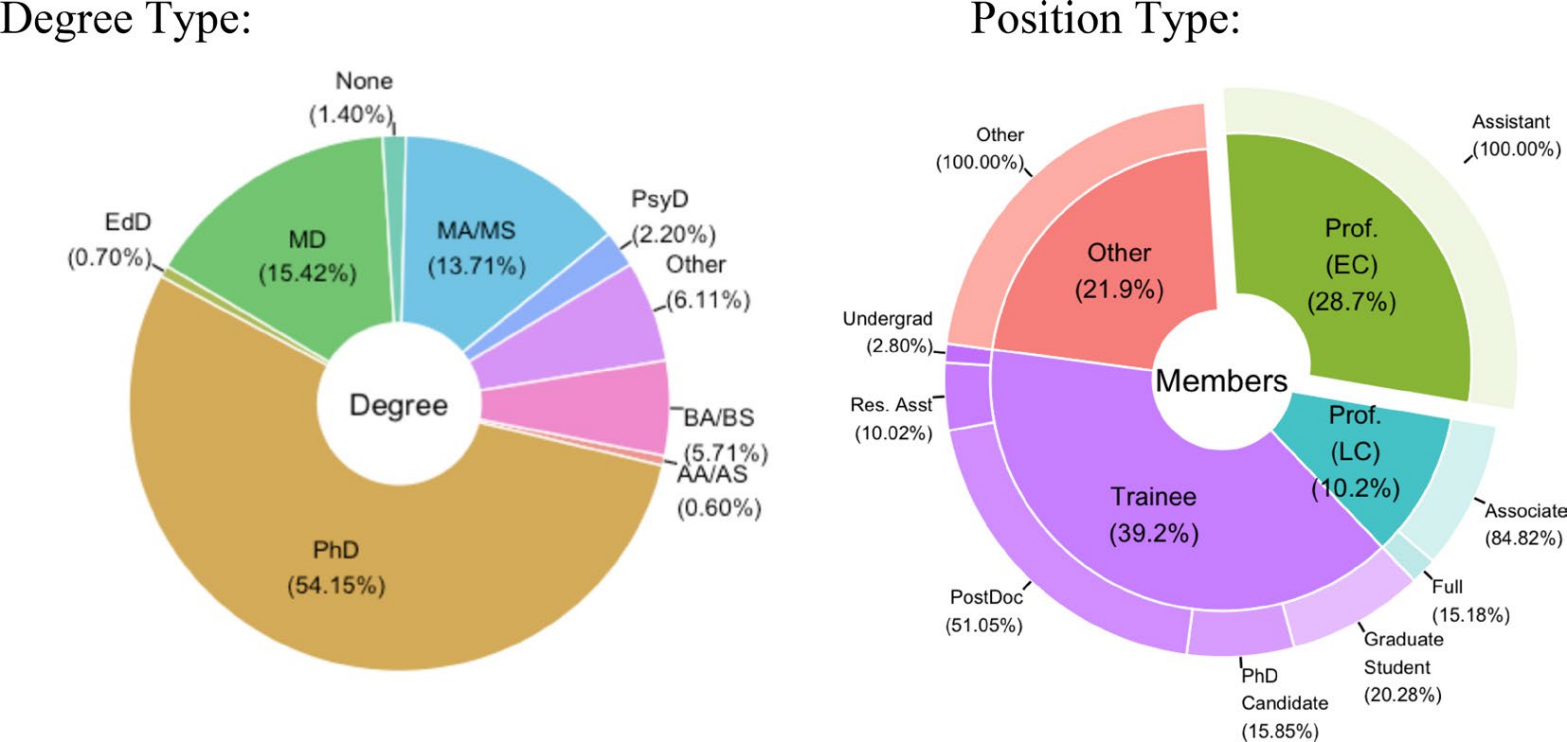
Early career INSAR members degree and positions. Degree Abbreviations: AA/AS = Associate of Arts/Science, BA/BS = Bachelor of Arts/Science, EdD = Doctor of Education, MA/MS = Masters of Arts/Science, MD = Doctor of Medicine, PharmD = Doctor of Pharmacy, PhD = Doctor of Philosophy, PsyD = Doctor of Psychology. Members were classified by terminal degree; an additional 5.2% of membership indicated “Other” (professional) certifications in addition to non‐Other degrees; 19.2% of MDs held an additional doctorate (PhD, PsyD, EdD, or PharmD). Position Abbreviations: EC = Early Career, LC = Later Career, Res. Asst = Research Assistant.

A large part of the INSAR ECC's focus is narrowing the gap between researchers from LMIC and upper‐income countries. Towards this aim, equitable early career support, particularly for researchers from geographically under‐represented countries is a priority across initiatives. The primary aim of this paper is to describe an equity‐forward model for the development and implementation of early career supports in the field of autism, where early career researchers from underrepresented communities are prioritized in program development (Figure 1).

## 2 Methods

### 2.1 Sample

**FIGURE 2:**
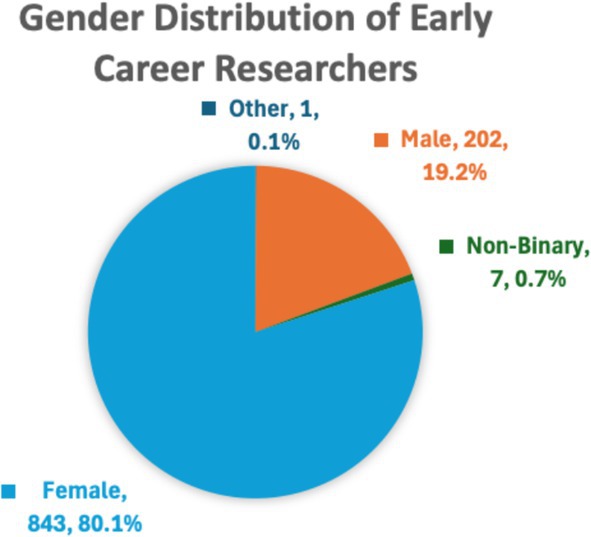
Gender distribution of early career researchers.

**FIGURE 3:**
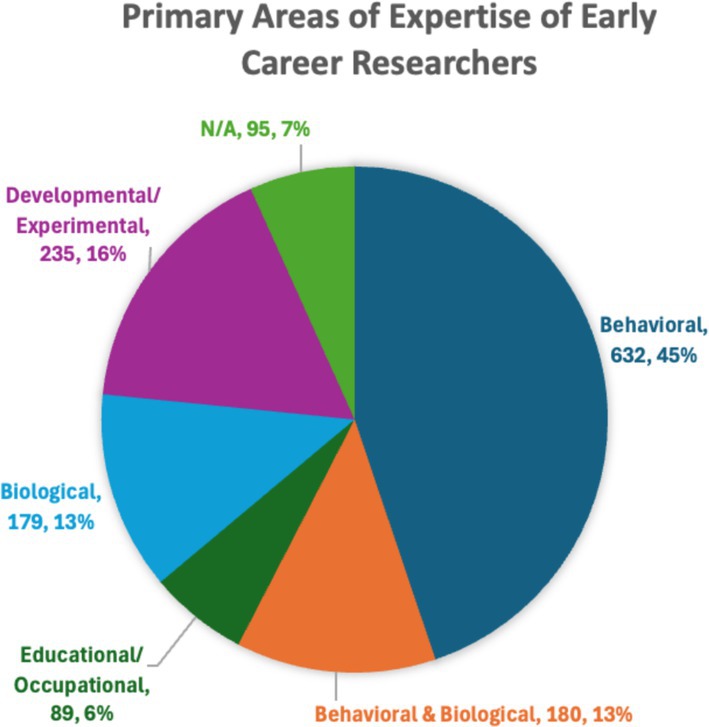
Primary areas of expertise of early career researchers.

Table 1 details the proportion of INSAR members who were early career (EC) members in 2018, 2019, and 2021 (in 2020 INSAR did not take place) and the proportion who were from LMIC based on income classification (World Bank 2021). Figures 2 and 3 demonstrate the breakdown of EC members by gender and areas of reported expertise. Examination of the data indicates that nearly 30% of INSAR members were EC researchers, and the vast majority were coming from high‐income countries (93%–97%). As more than a quarter of INSAR members are EC researchers, a need for initiatives specifically tailored for this career stage was identified. These were developed by the EC Committee with input from EC members during in‐person meetings and surveys conducted during the annual meetings in 2017 and 2018. Additionally, the lack of geographical diversity of EC INSAR members was identified as a priority based on the data reported in Table 1, leading to a focus on engagement, access, and mentorship for EC researchers across the globe. Specific initiatives and their outcomes are reported below. Of note, the opportunity to participate in the annual meeting virtually in 2021 did not result in increased participation of EC researchers in general or specifically those from LMIC (Table 2) suggesting that the simple provision of online access is insufficient to engage a geographically diverse membership.

**TABLE 1:** Geographic distribution of early career researchers.

	2018	2019	2021
EC members as a % (*n*) of total INSAR members	27.9% (496)	25.7% (656)	29.5% (573)
EC members from LMIC as a % (*n*) of total INSAR members	2.3% (9)	6.1% (43)	4.4% (25)

**TABLE 2:** Countries/territories represented in the early career mentoring initiative in 2018–2020.

Senior mentors	Early career mentees	Student trainee mentees
Australia	Australia	Australia
China	Canada	Belgium
India	Denmark	Canada
Taiwan	China	China
United Kingdom	Hong Kong	India
United States of America	India	Nigeria
	Israel	South Africa
	Malaysia	Switzerland
	Nigeria	Taiwan
	Puerto Rico	Uruguay
	Scotland	United Kingdom
	Turkey	United States of America
	Uruguay	Zimbabwe
	United Kingdom	
	United States of America	

### 2.2 Survey

In 2016, we requested to develop a survey assessing the needs of early career INSAR members. We created an 18‐item survey assessing the need of early career researchers within INSAR and interest in supporting programs around early career supports. Early Career was then defined as “Faculty or non‐academic professional autism researcher who is no longer a student or early‐stage post‐doc but still within 10 years of their terminal degree (advanced post‐docs beyond their first year included)” and the survey was sent out via INSAR to all members. We received 563 individual responses to the survey. Of those, approximately 277 identified as an early career member and over 25 distinct disciplines were represented. Many of the responders were post‐doctoral fellows (21.30%, *n* = 59) and tenure‐track assistant professors and had been in their current position for less than 5 years (17.69%, *n* = 49); the remainder of responders were a variety of non‐academic positions. The majority, or 95% (*n* = 254) of respondents are in support of INSAR creating and early career committee and 68% (*n* = 181) would like to join the committee. It should be noted that data was not collected on live participants at the INSAR 2017 Coffee Hour (Figure 1).

## 3 Results

### 3.1 INSAR Early Career Global Representative Initiative Program (GRIP)

The INSAR Early Career Global Representative Initiative Program (GRIP) started as a pilot program when it was launched in 2019 with the goal to expand and support the involvement of early career INSAR researchers from diverse backgrounds and geographical locations. The idea for the initiative originated from the first ECC INSAR event in 2017, which was attended by a geographically diverse group of INSAR attendees. During a brainstorming session focused on how to best meet the needs and expectations of EC researchers globally, several points were raised about the US‐centric nature of many INSAR initiatives at the time (e.g., workshops on developing grants for submission to specific US agencies). The same sentiment emerged during internal meetings among ECC members, leading to the decision of addressing the disconnection experienced by non‐US early career researchers between their priorities and the focus of most INSAR activities.

The GRIP initiative was conceptualized as a “two‐way” street, whereby the ECC would learn from geographically diverse EC researchers about their needs and priorities, and EC researchers would disseminate information about INSAR initiatives, informed by such needs, to their networks. Additionally, the initiative was designed to facilitate international networking, local capacity‐building, and regional expertise. This goal was addressed through the appointment, in 2019, of six “Global Representatives,” (i.e., INSAR members from diverse backgrounds and geographical locations: Australia, China, Denmark, India, South Africa, and Taiwan). Applicants for this role were selected among INSAR members based on several criteria, including their track record and goals in relation to autism research, their connection with local networks of autism clinicians and resources, and the degree to which their region of origin was under‐represented in INSAR activities.

The roles of the six Global Representatives include: (a) serving as the point of reference for other EC researchers from the same country at annual INSAR meetings, (b) networking with EC members in their countries to gather information on how they can be best supported in their training and professional development, (c) regional capacity building, and (d) liaising directly with the two ECC Global Outreach Officers (who are themselves the representatives from their own countries: Malaysia and the United States of America) to inform them of the needs and priorities specific to their regions of origin, as well as sharing ideas and resources in line with INSAR initiatives.

Although not all continents or geographical areas were represented, this initiative proved to be beneficial across multiple areas. For example, the global representatives indicated mentors from their regions to be involved in the Mentoring Initiative (see below), thus increasing the geographical representation of the initiative. Additionally, the global representatives had the opportunity to disseminate knowledge on that and other initiatives locally, thus expanding their global reach. Further, through the support of INSAR (e.g., use of INSAR social media to promote events) they expanded their regional networks and organized local events to discuss context‐specific training opportunities, funding mechanisms, and upcoming conferences relevant to EC researchers and clinicians. For example, the global representative from India created an event that involved a local leader in the field of autism to discuss the opportunities and challenges related to becoming a successful autism clinician and researcher in the region.

GRIP was later renamed from *Global Representative Initiative Pilot* to *Global Representative Initiative Program* after the successful 2 years pilot of the initiative. However, the aims and goals remained, as did the initial cohort of GRIP representatives. More recently, in 2024, a new cohort of GRIP representatives was selected to continue the legacy of GRIP and formulate expanded goals for subsequent generations of EC researchers. Future steps for the initiative involve expanding geographical representation from Africa and establishing representation from South America as well as organizing GRIP events when in‐person INSAR meetings resume, to facilitate interchange among global representatives and their respective networks. Additionally, GRIP hopes to form collaborations between GRIP regions and also establish links across career stages (student, early career, mid‐career and beyond), with the hope that the global outreach and connectivity of INSAR will be further enhanced via a new generation of autism researchers.

### 3.2 Research Rapid Rounds

The Early Career Committee disseminated a survey to EC members of INSAR and held a “coffee hour” networking session at the INSAR Annual Meetings in 2016 and 2017. The ECC analyzed the results and responses from EC members. The primary concern of EC members around the world was how to get their first independent grant; and subsequently how to get their second grant to sustain their career (e.g., promotion). The primary request from EC members to the EC Committee was to provide networking opportunities with funding agencies, journal editors, and senior researchers—all of whom could provide personalized advice and guidance on creating successful independent research programs and build attractive research portfolios for funding. In response to these requests of EC INSAR members, the Research Rapid Rounds annual event was created with the goal to foster a venue for EC researchers to get personalized, direct feedback from: funding agency program officers, journal editors, and senior investigators who often sit on study sections (collectively referred to as “reviewers”).

Research Rapid Rounds is an annual event held during lunch at the INSAR Annual Meeting. It was first held in 2018 and included 45 early career members who were matched with 15 expert reviewers from around the globe. Each EC researcher received the opportunity to “pitch” their grant proposal to a variety of reviewers with whom they were matched. Reviewers included (a) program officers for funding agencies; (b) reviewers of grants; (c) study section members; and (d) journal editors. The reviewers represented diverse fields and geographic areas that match the composition of respondents from EC surveys and previous INSAR meetings. The attendees were selected to be representative of global EC researchers. EC researchers were required to apply for the event and underwent rigorous review of their proposal in order to participate. The purpose of the application process was to ensure equitable access to the event for those with serious intention to submit a grant following the feedback process from reviewers. The inaugural event in 2018 included the following senior reviewers:

- Simon Baron‐Cohen (INSAR President; Cambridge Univ.).
- Sven Bolte (Karolinska Institutet).
- Tony Charman (King's College London).
- James Cusack (Autistica Director of Science).
- Geri Dawson (INSAR Past‐President; Duke Univ.).
- Petrus de Vries (University of Cape Town, South Africa).
- Tom Frazier (Autism Speaks Chief Science Officer).
- Hilde Geurts (INSAR VP; Univ. of Amsterdam).
- Lisa Gilotty (U.S. NIMH Program Director).
- Alycia Halladay (Autism Science Foundation Chief Science Officer).
- Shafali Jeste (INSAR Secretary; UCLA).
- Alice Kau (U.S. NICHD Program Director).
- Declan Murphy (King's College London).
- Liz Pellicano (Macquarie University, Australia).
- Diana Robins (INSAR Treasurer; Drexel Univ.).

The Research Rapid Rounds is now an annual fixture at each INSAR Annual Meeting since its inception in 2018. The event has hosted over 200 early career autism researchers and 60 senior reviewers. Over 77% of early career attendees indicated they would attend the event again, and 57% rated the event a 9 or 10 (out of 10). Table 3 reports feedback from EC attendees indicating specific aspects of the event that were particularly beneficial.

**TABLE 3:** Feedback from EC attendees of the Research Rapid Rounds event.

Having program officers as reviewers
Specific advice on my grant from well‐matched experts
Specific feedback on how to fit my ideas together and how best to frame them for grant
Advice on methodology
Getting to meet two seniors and a peer was very helpful
Having the opportunity to pitch it to a “live” person and receive feedback
The rapidness. I think a lot of attendees might say it wasn't long enough but it's not a rapid rounds for nothing. Every question and every comments needs to matter which makes it so helpful and productive
Having 2 reviewers with different perspectives
Time and perspective from funding bodies (e.g., XX) and the seniors did have some good advice
Meeting with program officer
Discussions with the mentors
1:1 time with an experienced reviewer/mentor
Getting individual feedback on my proposal from experts with experience applying and reviewing for similar mechanisms

### 3.3 Mentoring Initiative

The transition from trainee (e.g., postdoctoral fellow, psychology intern, and resident) to a first faculty position is a high‐risk period for attrition (Kupfer et al. 2009, 2016). Research demonstrates that early career faculty who receive strong mentorship at this point in their career are more productive and have higher levels of career satisfaction (Johnson et al. 2011; Sambunjak et al. 2006). Furthermore, successful junior mentoring programs have been associated with significant increases in faculty retention, with one program reporting that faculty who participated in their junior faculty mentoring program were 67% more likely to remain at their institution compared with junior faculty who did not participate in the program (Ries et al. 2009). In addition to this, surveys circulated by the INSAR ECC have repeatedly found that mentoring is ranked among the top needs of our early career colleagues.

In light of the pressing need for mentorship for early career researchers, in 2017, the ECC launched the INSAR ECC Mentoring Initiative, with the goal to build a tiered mentorship infrastructure within INSAR. The goals of the INSAR ECC Mentoring Initiative are to: (1) Use the network of expertise within INSAR to build the next generation of autism researchers through mentorship. (2) Build a tiered mentorship structure within INSAR by providing opportunities for early career members who do not have access to senior, established autism researchers at their home institution. The EC members then in turn provide mentorship, guidance, and advice on next steps (postdoc, faculty, etc.) for student/trainee (STC) members of the society. Through this tiered mentorship, we aimed to provide early career INSAR members with both mentorship from leaders in the field and the opportunity to mentor future early career scientists in the field.

Each year, the ECC mentoring initiative invited 10–15 senior researchers in the field to act as mentors for the early career mentees. These senior mentors were asked to (1) Mentor one early career member for 1 year. (2) Meet with early career member(s) at least once per quarter (a minimum of 4 times in the year). (3) Support the early career member(s) develop permanent products (e.g., manuscript, grant). (4) Allow early career member(s) to visit their lab/site/institution once during the year (at the mentee's expense, if possible). This was not a requirement for the mentees but an option and opportunity for them to learn from their mentors in person, if their budgets allow. In turn, the EC members were asked to (1) Mentor one ST member for 1 year. (2) Meet with ST member(s) at least every other month or as needed. (3) Collaborate with ST member(s) to develop permanent products (e.g., manuscript, training grant).

To apply for the ECC mentoring initiative, EC and ST members completed separate applications. The EC application asked each applicant to describe the type of mentorship they were currently receiving, the reason they needed a senior mentor through the mentoring initiative, the particular product that they wished to work on with their mentor if paired, and to rank and provide a reason for selecting their top three senior mentors. The application then asked about previous experience serving as a mentor to others, as well as metrics for success (number of manuscripts, grants, etc.). The ST application asked why they needed an early career mentor through the initiative, the goal they would like to work on with their mentor, and their overall career goals. Each application was rated independently by two individuals serving on either the INSAR ECC, the STC committee, or EC members who had participated in the prior year of the mentoring program (ST members only evaluated potential ST applicants) and both EC and ST applicants were rated using a scoring rubric that assessed four primary domains: (1) Need of the applicant. (2) Quality of the applicant/application. (3) Commitment to a career in autism research. (4) Overall fit with the program. Priority was given to EC members who did not have a primary mentor at their home institution, or EC members who needed a mentor in a different content area that was not available at their home institution. Priority was also given to non‐US Early Career members. The top three applicants per senior mentor were then sent to the mentor, who then ranked their choice based on fit with their research program. Once senior mentor‐EC pairs were made, the top ST mentees were paired to EC mentors based on areas of research interest.

In 2018, out of the 38 applications we received, we paired 10 senior mentors with 10 EC (4% of applicants) mentees from 7 countries. Those 10 EC mentees, plus the entire ECC were then paired with 16 ST mentees (out of 45 [36%] applicants) from 10 countries. As a result of these pairings, 12 papers were submitted and 5 published, 16 grants were submitted and 6 funded, and there were 2 INSAR presentations, including 1 first‐time attendee. When asked about the impact of the mentoring initiative, mentees shared:

My mentor was fantastic…In my first year as an assistant professor, I applied for a K23, R21, and an internal university grant. I was successful in being awarded all three. Thank you to INSAR for supporting this very important initiative—having my mentor's support has been instrumental to my successful first year as an Assistant Professor.

With the support of my mentor I was able to secure a one‐year postdoctoral fellowship grant from the NIH's Fogarty International Centre. I am currently doing a postdoc at the … My postdoctoral research aims to develop a culturally appropriate autism screening tool for low‐income and poor communities in middle‐income African countries.

In 2019, we recruited a new cohort of nine senior mentors who were then paired with 10 early career mentees (out of 36 applicants; 4% matched), and subsequently matched 10 student trainees (3% of the 29 applicants) to an EC mentor. In this new cohort, we had representation from eight different countries. Because the MI was impacted by the COVID‐19 pandemic, we extended the 2019–2020 MI an additional 6 months. We also introduced the concept of Individual Development Plans (IDP) to help structure the mentor‐mentee relationship. Each mentee was instructed to develop an IDP with their mentor within the first 3 months and send the IDP to the ECC Mentoring Initiative team for review. The IDP included structured questions on developing a Mission, Specific, Measurable, Achievable, Realistic, and Time‐bound goal (SMART), milestones, and assessing strengths and weaknesses. The IDP allowed for increase: communication between mentor and mentee, structure of the relationship, oversight, and opportunity to track outcomes and progress. Most importantly, the IDP has been shown to positively impact outcomes. Specifically, “Goal setting has a positive impact on performance and career outcomes.” The development of specific plans makes it more likely that goals are reached and “people who develop and implement strategies to pursue career‐specific goals achieve greater career success as measured by salary, promotions, and level of responsibility (Hobin et al. 2014).”

In 2021, we further developed the program by introducing a required mentorship webinar provided by the organizers of the Mentoring Initiative that all mentees were asked to attend. This introduced mentees to the principles of how to be a good mentee and how to get the most out of the mentoring relationship. This also allowed us to more explicitly describe expectations of mentees over the course of the Mentoring Initiative. In addition to this new webinar, thanks to collaboration with the GRIP initiative, we also were able to increase the representation of the senior mentors across the globe.

## 4 Limitations of ECC Initiatives

Expanding and implementing the ECC initiatives faced several challenges, including technological access limitations, time zone differences, and varying levels of institutional support. Limited internet connectivity or access to necessary platforms can create disparities in participation, while global time zone differences complicate real‐time engagement in virtual programs. Additionally, early‐career professionals in under‐resourced institutions may lack the necessary support to fully benefit from ECC initiatives. To overcome these barriers, the ECC has relied on virtual opportunities as well as those offered at the annual meeting in addition to asynchronous opportunities for those in the Mentoring program, ensuring accessibility regardless of location. GRIP focused on more regional collaborations to create localized support networks that address time zone constraints and resource disparities. Since many of our initiatives were developed with equity in mind, access was also top of mind and built in where possible to better support early‐career professionals worldwide.

## 5 Call to Action

Investing in early‐career professionals is essential for building a skilled, diverse, and sustainable workforce. The initiatives led by the ECC play a critical role in supporting the retention and professional growth of early‐career researchers by providing structured opportunities for mentorship, networking, and skill development. By addressing common barriers such as limited institutional support, technological access, and time zone constraints, these initiatives help them stay engaged and motivated in their fields. Additionally, targeted programs focused on career development, research training, and collaboration enhance skill‐building, making early‐career researchers more competitive for promotions and long‐term career advancement. Strengthening the support system for early‐career professionals not only benefits individuals but also has larger implications for workforce development, as a well‐supported pipeline of emerging researchers contributes to innovation, productivity, and moving the field forward. When organizations invest in early‐career researchers, they have the potential to cultivate future leaders who will drive progress in their respective fields, fostering a more dynamic and resilient scientific landscape.

Our program for establishing support around challenges experienced during the early career period may serve as a model for other scientific fields in the future. This critical career phase often coincides with personal life milestones (e.g., parenting young children, supporting aging parents) and changes in one's formal mentoring support. Career development research demonstrates that many successful researchers fall through the cracks following during the early career stages (Kupfer et al. 2009), especially those with inadequate internal resources (Crews et al. 2020). However, enhanced mentoring supports have demonstrated effectiveness for this career stage (Kupfer et al. 2016). Further significant variability exists in supports across the globe in autism career development. While the field of autism has expanded greatly since the inception of INSAR in 2001, as evidenced by large multisite longitudinal collaborations and a significant increase in the number of researchers, we must now focus on developing models to support equitable access to opportunities in the field. Our model demonstrates that when prioritizing those in need of more support, the entire field benefits, including the quality of research. The focus on culturally sensitive and geographically representative early career researchers is critical to encourage the study of autism in underrepresented regions and cultures. This, in turn, has the potential to bring diverse perspectives and new knowledge in the field that can counteract the biases ingrained within the Western autism research “establishment.” In 2006, INSAR invested in the student and trainee population, which subsequently resulted in significant early career member growth. This continued expansion of early career researchers is an unprecedented opportunity that must be supported by inclusive, diverse, and equitable support.

## Author Contributions

Dr. Michele E. Villalobos conceptualized and designed the study, collected data, carried out the initial analyses, drafted the initial manuscript, and critically reviewed and revised the manuscript. Dr. Giacomo Vivanti carried out initial analyses, reviewed the initial analysis, made substantial revisions to the final manuscript. Drs. Subhashini Jayanath, Kimberly Carpenter, Mark D. Shen, and Frederick Shic drafted the initial manuscript, and critically reviewed, and revised the manuscript. Dr. Frederick Shic helped create the figures in the manuscript. Dr. Jill Locke co‐designed the study and supervised manuscript conceptualization and critically reviewed and revised the manuscript. All authors approved the final manuscript as submitted and agreed to be accountable for all aspects of the work.

## Conflicts of Interest

The authors declare no conflicts of interest.

## Data Availability

The data that support the findings of this study are available from the corresponding author upon reasonable request.
